# Biotransformation of Cranberry Proanthocyanidins to Probiotic Metabolites by *Lactobacillus rhamnosus* Enhances Their Anticancer Activity in HepG2 Cells *In Vitro*

**DOI:** 10.1155/2019/4750795

**Published:** 2019-06-17

**Authors:** H. P. Vasantha Rupasinghe, Indu Parmar, Sandhya V. Neir

**Affiliations:** ^1^Department of Plant, Food, and Environmental Sciences, Faculty of Agriculture, Dalhousie University, Truro, Nova Scotia, Canada; ^2^Department of Pathology, Faculty of Medicine, Dalhousie University, Halifax, Nova Scotia, Canada

## Abstract

This study was designed to unravel the role of *Lactobacillus rhamnosus* in the bioconversion of cranberry proanthocyanidins and cytotoxicity of resulting metabolites to hepatocellular carcinoma HepG2 cells. Crude (CR) and flavonol+dihydrochalcone- (FL+DHC-), anthocyanin- (AN-), proanthocyanidin- (PR-), and phenolic acid+catechin- (PA+C-) rich fractions were subjected to fermentation with *L. rhamnosus* at 37°C for 12, 24, and 48 h under anaerobic conditions. The major metabolites produced by bioconversion of polyphenols were 4-hydroxyphenylacetic acid, 3-(4-hydroxyphenyl)propionic acid, hydrocinnamic acid, catechol, and pyrogallol. Furthermore, cytotoxicity of the biotransformed extracts was compared to their parent extracts using human hepatocellular carcinoma HepG2 cells. The results showed that PR-biotransformed extract completely inhibited HepG2 cell proliferation in a dose- and time-dependent manner with IC_50_ values of 47.8 and 20.1 *μ*g/mL at 24 and 48 h, respectively. An insight into the molecular mechanisms involved revealed that the cytotoxic effects of PR at 24 h incubation were mitochondria-controlled and not by proapoptotic caspase-3/7 dependent. The present findings suggest that the application of a bioconversion process using probiotic bacteria can enhance the pharmacological activities of cranberry proanthocyanidins by generating additional biologically active metabolites.

## 1. Introduction

Primary liver cancer, also known as hepatocellular carcinoma (HCC), is the fifth common cancer and the third leading cause of cancer mortality in the world [1]. HCC is caused in a milieu of oxidative stress and inflammation, with its pathogenesis represented by the production of cytokines and chemokines, generation of free radicals, such as reactive oxygen and nitrogen species, viral infections, hepatitis, hepatic cirrhosis, and hepatocarcinogens. From the very few clinically relevant therapeutic options available of HCC, sorafenib (Nexavar™), a vascular endothelial growth factor receptor tyrosine-kinase inhibitor, is the only approved drug. However, clinical administration of sorafenib is challenged by the low survival rate and several adverse effects in patients including hematological toxicity [2]. Therefore, a need for alternative anticancer strategies holds importance for clinical and experimental oncology.

Plant polyphenols are known to possess strong antioxidant properties and have been shown to exhibit anti-inflammatory, antiproliferative, and proapoptotic properties, suggesting their role as chemopreventive agents [3]. However, the use of polymeric polyphenols such as proanthocyanidins is limited in chemoprevention because of their poor bioavailability in the human body. The bioavailability and physiological functions of proanthocyanidins are largely influenced by their molecular weight, structural complexity, digestibility, intestinal absorption, metabolism, and gut microbiota. Structural modification, nanoencapsulation, and biotransformation are some of the strategies to combat the issue of low bioavailability of polyphenols [4]. Biotransformation of polyphenol-rich foods can be performed by incubating with microorganisms, especially probiotics which enable deglycosylation, ring fission, dehydroxylation, demethylation, lactonization, aromatic hydroxylation, reduction of carbon-carbon double bonds, or decarboxylation [5, 6], converting some polyphenols into more bioavailable and/or bioactive forms than their original forms [7].

Cranberry pomace is a by-product of the cranberry juice-processing industry, which is rich in numerous phenolic compounds with potential health benefits. Some of the major phenolics present in cranberry pomace include anthocyanins, proanthocyanidins, flavonols, phenolic acids, and flavan-3-ols [8]. Cranberry press cake-based flavonoid extract has been demonstrated to exhibit significant effects against various cancer cell lines including the prostate (LNCaP), melanoma (MDA-MB-435), malignant melanoma (SK-MEL-5), colon (HT-29), lung (DMS114), and brain (U87) [9]. Our recent investigation has shown that only small molecular weight phenolic acid-rich fractions were able to impart antiproliferation activity at low concentrations in human HCC cell line HepG2 as reflected from their IC_50_ values [8].

*Lactobacillus* species is one of the predominant members of the intestinal microflora, and some strains have been characterized as probiotics. Species of *Lactobacillus* possess *β*-glucosidase activity and participate in the hydrolysis of plant *β*-glycosides [10]. *Lactobacillus plantarum* has been shown to metabolize phenolic acids and esters of phenolic acids by the activities of tannase [11], feruloyl esterase [12], phenolic acid decarboxylase, and phenolic acid reductase [13]. These activities of Lactobacilli may contribute to the release of phenolic acids bound to insoluble cell wall material, particularly protocatechuic and *p*-hydroxybenzoic acids. However, little is known about flavonoid biotransformation by Lactobacilli and their resulting anticancer properties [6]. Herein, we investigated the capability of *L. rhamnosus* to biotransform five different polyphenol-rich fractions from cranberry pomace and explored their anticancer activity against HCC using HepG2 cells in comparison to sorafenib. In addition, their mechanistic approach to the cytotoxicity has also been presented through ATP depletion and caspase-3 activity.

## 2. Materials and Methods

### 2.1. Chemicals and Standards

The liquid chromatography standards used for the study were obtained as follows: quercetin-3-*O*-rhamnoside and quercetin-3-*O*-galactoside were from Indofine Chemical Co. (Hillsborough, NJ, USA); quercetin-3-*O*-rutinoside, (-)-epicatechin, (+)-catechin, epigallocatechin (EGC), epicatechin gallate (ECG), epigallocatechin gallate (EGCG), and procyanidin B1 and B2 were from ChromaDex (Santa Ana, CA, USA); and cyanidin-3-*O*-galactoside was obtained from Extrasynthese (Paris, France). High-performance liquid chromatography (HPLC) grade methanol, acetonitrile, and formic acid; quercetin-3-*O*-glucoside, quercetin, phloridzin, myricetin, and phenolic acids (caffeic acid, ferulic acid, gallic acid, protocatechuic acid, chlorogenic acid, *trans*-cinnamic acid, p-coumaric acid, isoferulic acid, *p*-hydroxybenzaldehyde, p-hydroxybenzoic acid, vanillic acid, and vanillin); syringic acid, gallic acid, homogentisic acid (2,5-dihydroxyphenylacetic acid), sinapic acid (3,5-dimethoxy-4-hydroxycinnamic acid), DMB propionic acid (3–20,50-dimethoxybenzoylpropionic acid), p-hydroxybenzaldehyde, p-hydroxybenzoic acid, vanillic acid, and vanillin; syringic acid (3,5-dimethoxy-4-hydroxybenzoic acid), homogentisic acid (2,5-dihydroxyphenylacetic acid), sinapic acid (3,5-dimethoxy-4-hydroxycinnamic acid), p-coumaric acid (trans-4-hydroxycinnamic acid), DMB propionic acid (3–20,50-dimethoxybenzoylpropionic acid), and dimethyl sulfoxide were obtained from Sigma-Aldrich (Mississauga, ON, Canada). The remaining chemicals were obtained from Fisher Scientific (Ottawa, ON, Canada).

### 2.2. Isolation of Bioactive Fractions from Cranberry Pomace

The cranberry pomace was collected from a commercial cranberry juice manufacturer (Cranberry Acres Farm, Aylesford, NS, Canada). Immediately after juice processing, the excess water was drained and within 3 h, the cranberry pomace was transported in plastic containers to the laboratory facility and stored in a freezer (-20°C). Five hundred grams of frozen cranberry pomace was ground with 2 L extraction solvent (70% acetone : 29.9% water : 0.1% acetic acid by *v* : *v* : *v*) using a Waring glass blender (Model CAC32, Fisher Scientific (Ottawa, ON, Canada) for 3 min. The slurry was then subjected to sonication for 30 min × 3 at 30°C with 10 min intervals in between every 15 cycles to avoid increase in temperature. The suspension was then passed through eight layers of cheesecloth followed by vacuum filtration using Fisherbrand P8 filters. The crude extract was then rendered solvent free using the rotary vacuum evaporation system (Rotavapor HR-200; Buchi, Flawil, Switzerland) at 37°C until completely dry. A representative sample was taken and named as crude (CR) extract. The concentrated extract was dissolved into 120 mL of 50% ethanol-water mixture.

**Figure 1:**
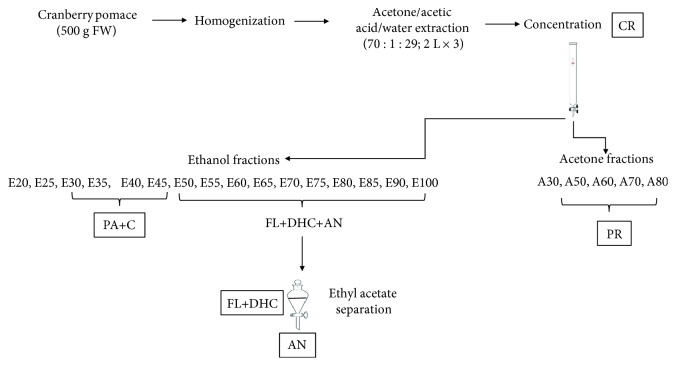
Process flow chart for extraction and fractionation of cranberry pomace (CP). PA: phenolic acids; DHC: hihydrochalcones; AN: anthocyanins; PR: proanthocyanidins; FL: flavonols.

Flash chromatography with a sorbent (Sorbent SP207-05 Sepabeads resin brominated styrenic adsorbent; particle size 250 mm, surface area 630 m^2^/g; Sorbent Technologies, Atlanta, GA, USA) was used to fractionate the concentrated cranberry pomace extract as described above. A detailed diagram of the process is presented in Figure 1. The crude extract was loaded onto a chromatography column (3.86 × 45 cm, Sati International Scientific Inc., Dorval, QC, Canada) that contained 400 g of adsorbent and had been conditioned with deionized water and equilibrated in 50% ethanol-water mixture. After loading the extract, the column was immediately washed with water until the sugars were removed up to a Brix value of <0.1%, as measured by a digital hand-held refractometer. The phenolic compounds retained in the column were eluted using a step gradient of ethanol (1 L per elution). The phenolic acid, flavonol, flavan-3-ol, and anthocyanin-rich fractions were eluted with 20-100% ethanol with an increment of 5% ethanol per elution. Then, a step gradient of acetone (1 L per elution) was carried out to elute oligomeric and polymeric proanthocyanidin-rich fractions. These elutions were carried out using 20%, 30%, 50%, 60%, 70%, 80%, and 100% acetone, pooled together, and evaporated to a completely dry powder that was labeled as PR. The fractions rich in phenolic acids and catechins were combined and evaporated to produce PA+C extract. A liquid-liquid separation method using ethyl acetate and water was employed for the separation of anthocyanins (AN) from fractions rich in flavonols and dihydrochalcones (FL+DHC). Eluates were concentrated to completely dried extracts using a rotary evaporator at 40°C.

### 2.3. Bacterial Strain and Culture Conditions

A strain of *Lactobacillus rhamnosus* (ATCC 9595) was cultivated in De Man, Rogosa, and Sharpe broth (MRS; BD, Becton, Dickinson, and Co., Sparks, MD, USA) at 37°C for 24 h in anaerobic jars (Gas-Pack, AnaeroGen; Oxoid, Nepean, ON, Canada). Bacterial growth was carried out in triplicate wells of sterile 96-well microplates with a lid, containing 300 *μ*L of MRS broth with or without different concentrations of extracts. Wells were inoculated (1%; about 10^7^ colony-forming units/mL) with a fresh culture of the strain incubated in MRS broth at 37°C for 16 h. Appropriate controls were used by incubating noninoculated MRS broth, CP extracts (blank), and MRS broth inoculated with the strain (growth control). Incubations were carried out at 37°C for 48 h under shaking of 70 rpm. Growth was monitored by recording the OD_600_ variations after 6, 12, 24, and 48 h of incubation at 37°C using a BMG FLUOstar Optima microplate reader.

### 2.4. Microbiological Analysis and pH Measurement

A 10 mL fresh broth was inoculated overnight with an *L. rhamnosus* culture at a concentration level of McFarland 0.5 standard. Dried CP extracts were added to the culture media to give final concentrations of 1 mg/mL. The cultures were shaken well and incubated according to the above-mentioned conditions. Bacterial growth curves were determined by reading the sample OD_625_ at various time points (0-48 h). The pH value of each fermented sample (10 mL) was measured after 0, 24, and 48 h of fermentation using a pH meter (Table 1).

### 2.5. Biotransformation of CP Extracts

Cultures used to follow catabolism of CP extracts by *L. rhamnosus* were performed in MRS broth by scaling volumes up to 100 mL using 125 mL sterile Erlenmeyer flasks. Solutions of extracts (0.25, 0.5, and 1 mg/mL) were prepared in the bacterial medium and sterile filtered before use. During incubation (37°C) with a previously grown 24 h culture, samples were taken at desired intervals of 12, 24, and 48 h. Cell pellets were sonicated for 30 min to lyse cells and release any phenolics that had been adsorbed on or in the bacteria cells. Cell lysates were centrifuged at 4900 ×*g* for 10 min, and supernatants were separated, which were used to extract phenolic metabolites from media using ethyl acetate (EA) partition technique. Briefly, equal volumes of supernatant and EA were mixed in a separation funnel and allowed to stand for 24 h until complete phase separation. Then, the EA and aqueous layers were separately collected. The EA layer was evaporated using a rotary vacuum evaporator, re-dissolved in 80% methanol, filtered through 0.22 *μ*m nylon filters, and kept at −20°C until further analysis.

### 2.6. Qualitative Analysis Corresponds to the Identification of Individual Phenolic Compounds in CP Fractions

Total phenolic content (TPC) was determined using the Folin-Ciocalteu method as described by Singleton and Rossi [14] and modified by Rupasinghe et al. [15]. The results are expressed in mg gallic acid equivalents (GAE)/L. Total anthocyanin contents (TAC) were determined using the pH differential method (AOAC method 2005.02) as previously described by Ratnasooriya et al. [16], and their concentration was expressed as mg cyanidin-3-*O*-glucoside equivalence (C3G) per 100 g FW using a molar extinction coefficient (*ε*) 28,000 and molecular weight (MW) 484.8 for C3G. Total flavonoid content (TFC) was measured using the aluminum chloride method as described previously [17], and total proanthocyanidin content (TPr.C) was assessed using the dimethyl cinnamaldehyde method [18]. The results are expressed as mg quercetin equivalents (QE)/L for TFC and mg catechin equivalents (CE)/L for TPr.C.

### 2.7. Determination of Phenolic Compounds and Their Metabolites

Out of each extract, 10 mg was weighed out and dissolved in 10 mL methanol, followed by required dilution, filtration through a 0.22 *μ*m nylon filter, and placing into amber vials. An ultrahigh performance liquid chromatography (UHPLC) (Model H-class system, Waters, Milford, MA, USA) equipped with an Acquity UHPLC BEH C_18_ column (2.1 × 100 mm, 1.7 *μ*m) (Waters, Milford, MA, USA) was used for analysis. For the analysis of nonanthocyanin phenolics, gradient elution was carried out with 0.1% formic acid in water (solvent A) and 0.1% formic acid in acetonitrile (solvent B), with the flow rate of 0.2 mL/min and an injection volume of 2.0 *μ*L. A linear gradient profile was used with the following proportions of solvent A applied at time *t* (min): (*t*, A%): (0, 94%), (2, 83.5%), (2.61, 83%), (2.17, 82.5%), (3.63, 82.5%), (4.08, 81.5%), (4.76, 80%), (6.75, 20%), (8.75, 94%), and (12, 94%). The analysis of anthocyanins was performed as described below; the mobile phases were 5% (*v*/*v*) formic acid in water (solvent A) and 5% (*v*/*v*) formic acid in methanol (solvent B). The linear gradients used were as follows: (*t*, A%): (0, 10%), (8, 30%), (17, 40%), (19, 40%), (20, 10%), and (22, 10%).

MS-MS analysis was performed with a Micromass Quattro micro API MS/MS system, which is controlled by a MassLynx V4.1 data analysis system (Micromass, Cary, NC, USA) as described by Bhullar and Rupasinghe [18]. Electrospray ionization in negative ion mode (ESI-) was used for the ionization of the flavonol and flavan-3-ol compounds. The mass spectrometry conditions included capillary voltage of 3000 V with nebulizing gas (N_2_) at a temperature of 375°C. Electrospray ionization in positive mode (ESI+) was used for the analysis of anthocyanins. The mass spectroscopy conditions used were a capillary voltage of +3500 V with nebulizer gas at 375°C and a flow rate of 0.35 mL/min. The cone voltage (25-50 V) was optimized for each compound. Individual samples were identified using a multiple reaction monitoring mode and specific precursor-product ion with quantified calibration curves generated by external standards. For the analysis of phenolic metabolized produced by *L. rhamnosus*, ESI- mode (as described above) and single-ion monitoring (SIM) mode were used as follows: *m*/*z* 183 for benzoic acid, *m*/*z* 179 for caffeic acid, *m*/*z* 353 for chlorogenic acid, *m*/*z* 163 for p-coumaric acid, *m*/*z* 193 for ferulic acid and isoferulic acid, *m*/*z* 149 for hydrocinnamic acid, *m*/*z* 151 for 4-hydroxyphenylacetic acid, *m*/*z* 165 for 3-(4-hydroxyphenyl)propionic acid, *m*/*z* 117 for succinic acid, *m*/*z* 197 for syringic acid, and *m*/*z* 153 for protocatechuic acid.

### 2.8. Biological Activity Determination

#### 2.8.1. Cell Lines and Culture Conditions

Human hepatocellular carcinoma (HepG2) cells was purchased from the American Type Culture Collection (ATCC HB-8065, Rockville, MD, USA) and cultured as recommended by the ATCC as described by Nair et al. [19]. HepG2 cells were grown in Eagle's modified minimum essential media (EMEM) supplemented with 10% FBS (FBS; ATCC, Rockville, MD, USA) and 1% penicillin-streptomycin (ATCC, Rockville, MD, USA). Cells were maintained at 37°C in an incubator under 5% CO_2_/95% air atmosphere at above 85% relative humidity constantly. Cells were counted using a hemocytometer (Bright-Line Hemacytometer, Sigma-Aldrich, Mississauga, ON, Canada) and were plated according to the number of cells for each experiment in a 6-, 24-, or 96-well format for 24 h prior to the addition of test compounds. All the test samples were solubilized in sterile filtered DMSO (<0.5% in the culture medium) before addition to the culture media. Control cells were also run in parallel and subjected to the same changes in media with <0.5% DMSO.

#### 2.8.2. Antiproliferation Activity before and after Bioconversion

HepG2 cells (5 × 10^3^ cells/100 *μ*L/well) were seeded in a sterile flat bottom 96-well plate (BD Biosciences, Mississauga, ON, Canada) and stabilized by incubation for 24 h at 37°C in a humidified incubator containing 5% CO_2_ (VWR, Mississauga, ON, Canada) [19]. CP metabolites after 24 h bioconversion were used for their ability to inhibit human liver cancer cell proliferation and compared with their parent fractions (0 h). These samples were selected based on their polyphenol metabolite characterization as analyzed by UPLC/MS. From each extract (CR, AN, FL+DHC, and PA+C), 10 mg was weighed out and dissolved in DMSO to produce a stock solution of 5000 mg/L. PR 0 h, PR 24 h, 3-(4-hydroxyphenyl)propionic acid, 4-hydroxyphenylacetic acid, and sorafenib were dissolved in DMSO and diluted in media, and 100 *μ*L of each treatment was added to each well, each treatment in three replications. Thereby, cells were exposed to various concentrations (10, 50, 100, 250, and 500 *μ*g/mL) of each treatment. Controls consist of cells with media containing DMSO (<0.5%), and blank wells contained media with no cells. After 24 and 48 h of test compound incubation, 20 *μ*L of the MTS reagent in combination with the electron-coupling agent, phenazine methosulfate, was added to the wells and cells were incubated in a humidified CO_2_ incubator for 3 h. Absorbance at 490 nm (OD_490_) was monitored with a plate reader (FLUOstar Optima, BMG Labtech, Durham, NC, USA) to obtain the number of viable cells relative to the control population. Percentage of viability in the test compound-treated cells is expressed as a percentage compared to the control (<0.5% DMSO). Data are expressed as mean values ± SD and obtained from thee different experiments against each cell line (*n* = 3 per plate per time point).

#### 2.8.3. ATP Luminescent Cell Viability Assay

The CellTiter-Glo® Luminescent Cell Viability Assay Kit (Promega, Madison, WI, USA) was used as a homogeneous method to determine the number of viable cells in culture was based on a quantification of ATP levels [19]. HepG2 cells (5 × 10^3^ cells/100 *μ*L/well) were seeded on opaque-walled 96-well black plates (BD Biosciences, San Jose, CA, USA) and allowed to attach for 24 h. Cells were then exposed to various concentrations (10, 20, 50, and 100 *μ*M) of PR 0 h, PR 24 h, 3-(4-hydroxyphenyl)propionic acid, 4-hydroxyphenylacetic acid, and sorafenib or DMSO (control) in the EMEM medium for 24 h. CellTiter-Glo® Reagent (100 *μ*L) was added to each well and mixed for 2 min on an orbital shaker to induce cell lysis. After 10 min of incubation at room temperature, luminescence was recorded using the above-described microplate reader.

#### 2.8.4. Caspase 3/7 Activity Assay

HepG2 cells (5000 cells/100 *μ*L/well) were seeded in white-walled 96-well plates and treated with 50 and 100 *μ*M of PR 0 h, PR 24 h, 3-(4-hydroxyphenyl)propionic acid, 4-hydroxyphenylacetic acid, and sorafenib or DMSO (control). After 24 h of incubation in 37°C/5% CO_2_ humidified incubator, 100 *μ*L of Caspase-Glo® 3/7 reagent was added to each well of a white-walled 96-well plate containing 100 *μ*L of blank, negative control cells, or treated cells in the culture medium. After mixing the contents of wells using a plate shaker for 30 s, plates were incubated at room temperature for 3 h. Luminescence was measured using the previously described microplate reader.

### 2.9. Experimental Design and Statistical Analysis

All analyses were conducted twice for three replicates per experiment. The data were analyzed using the Statistical Analysis System software (SAS Institute Inc., Cary, NC, USA). The general linear model (GLM) procedure was used to evaluate the main effect of treatment. Tukey's studentized test was used to compare the means among treatments at a *p* value of 0.05.

## 3. Results and Discussion

### 3.1. Isolation and Quantification of CP Fractions

**Figure 2:**
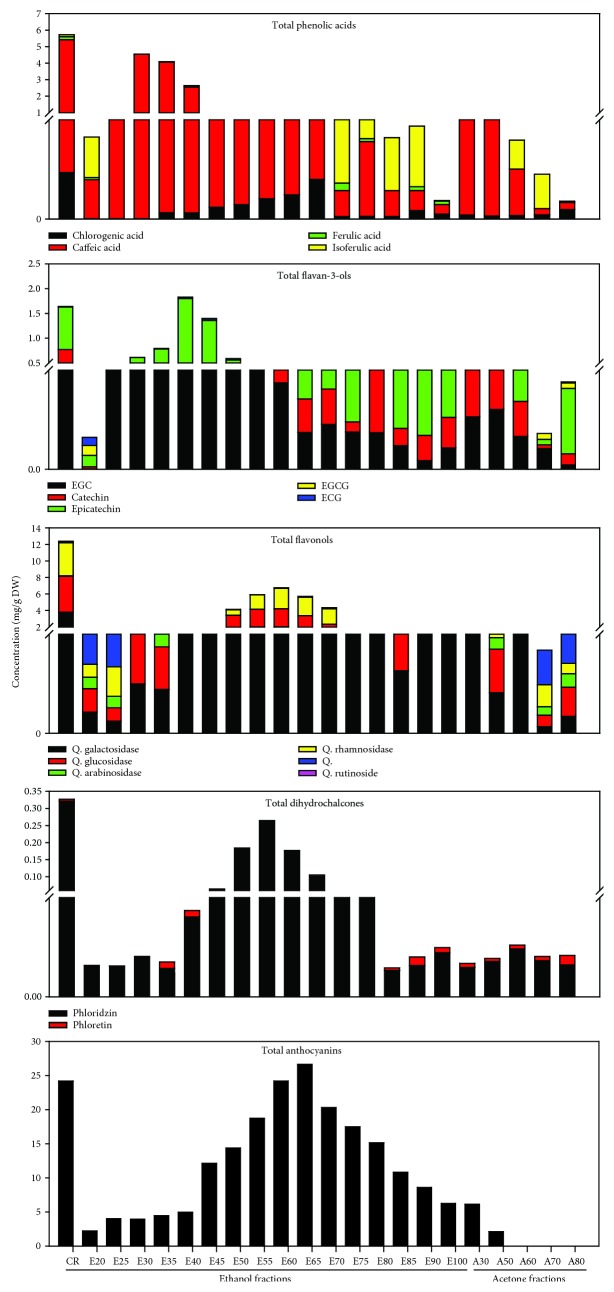
Characterization of different cranberry pomace fractions by UPLC-ESI-MS. Flavan-3-ols are represented by the sum of catechin, epicatechin, EGC, ECG, and EGCG. Phenolic acids are a sum of chlorogenic, ferulic, and caffeic acids. Flavonols are a sum of quercetin glucoside and galactoside; Dihydrochalcones represent the sum of phloridzin and phloretin. Anthocyanins represent cyanidin-3-*O*-glucoside, the most predominant anthocyanin.

The phenolic characterization of CP is presented in Figure 2. Based on some of the major phenolics present in CP, different fractions were pooled accordingly to obtain five major fractions: phenolic acid and catechin- (PA+C-), flavonol+dihydrochalcone- (FL+DHC-), anthocyanin- (AN-), and proanthocyanidin- (PR-) rich fractions and crude (CR) fraction. Fractions E25-E45 exhibited the highest amounts of phenolic acids (sum of chlorogenic acid, ferulic acid, isoferulic acid, and caffeic acid) and flavan-3-ols (sum of EGC, catechin, epicatechin, EGCG, and ECG) and hence were pooled together to obtain PA+C. E50-E90 displayed the highest amount of flavonols (sum of quercetin aglycones and its various glycosides including galactoside, glucoside, rhamnoside, rutinoside, and arabinoside) and dihydrochalcones, anthocyanins (cyanidin-3-*O*-glucoside), and dihydrochalcones (phloridzin and phloretin). In order to separate anthocyanins, further liquid-liquid extraction was carried out using the ethyl acetate-water solvent system. The ethyl acetate fraction exhibited the highest amount of flavonols and dihydrochalcones (FL+DHC), while the water extract contained most of the anthocyanins (AN). All acetone fractions were pooled together to obtain PR. The CR showed the presence of almost all the analyzed phenolic compounds at moderate concentration levels. While the AN was rich in cyanidin-3-*O*-glucoside, it also had a small quantity of flavonols, especially quercetin galactoside. PR fraction was rich in proanthocyanidins, as determined by the DMAC analysis. The FL+DHC fraction exhibited the high amounts of quercetin glycosides, especially the galactosides and rhamnosides along with phloridzin as dihydrochalcone. In addition, the FL+DHC also contained some low molecular weight phenolic acids such as ferulic acid and isoferulic acid. The PA+C consisted of major phenolic acid and flavan-3-ols, including catechin and epicatechin, and minor amounts of EGC, EGCG, and ECG.

### 3.2. Bacterial Growth in the Presence of CP Fractions

The concentration of 1000 mg/L was used for all three extracts as the growth of *L. rhamnosus* was not inhibited significantly by any of the CP extracts at this concentration (Table 2). This could be due to the growth promotion of probiotic bacteria such as *Lactobacilli* by polyphenols. Whereas all the polyphenol-rich extracts were effective, the concentration level of 250 mg/L stimulated the greatest percentage increases (Table 2). At the end of 24 h using 1000 mg/L, the percentage growth of *L. rhamnosus* was not significantly different from those in the control, except in the AN extract. However, concentration levels above 1000 mg/L significantly affected the bacterial growth. In addition, it was found out that different extracts exhibited varying sensitivity towards *L. rhamnosus*. *L. rhamnosus* was capable of growing in the presence of different CP extracts, yet the growth was being affected in a dose-dependent fashion (Table 2). The results showed a progressive decrease in maximal OD_625_ from a concentration of 250 to 1500 mg/L. The decrease in the growth rate was most evident in the presence of AN extract, although the extracts did not completely inhibit the growth of *L. rhamnosus*. The CR did not exhibit any significant inhibition at the tested concentration levels. This was in line with a previous study where cranberry extracts produced no major inhibition at concentrations of 250-1000 mg/L against *L. plantarum* [20]. The same study also showed cranberry-specific proanthocyanidin A2 as a growth promoter of *L. plantarum*, which was found to be true for *L. rhamnosus* in the present study. Interestingly, the AN and PA+C were found to inhibit *L. rhamnosus* by 35% and 31%, respectively, at a concentration of 1500 mg/L. For PA+C, this could be due to the presence of galloylated flavanols having high hydrophobicity and thus high affinity for the phospholipid cell membrane [21].

### 3.3. Polyphenol Biotransformation by *L. rhamnosus*

**Figure 3:**
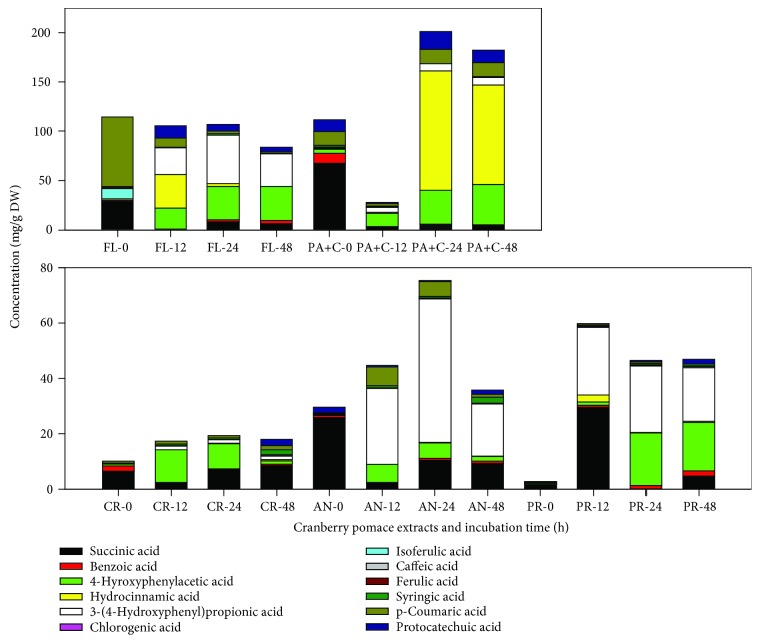
Changes in concentration of different phenolic acids as a result of bioconversion of cranberry proanthocyanidins using *L. rhamnosus* during the incubation. Data presented the mean values of *n* = 3 with respect to the medium control. CR: crude; AN: anthocyanin; PR: proanthocyanidin: FL: flavonol; PA+C: phenolic acid+catechin. 0, 12, 24, and 48 are the incubation time (hours).

**Figure 4:**
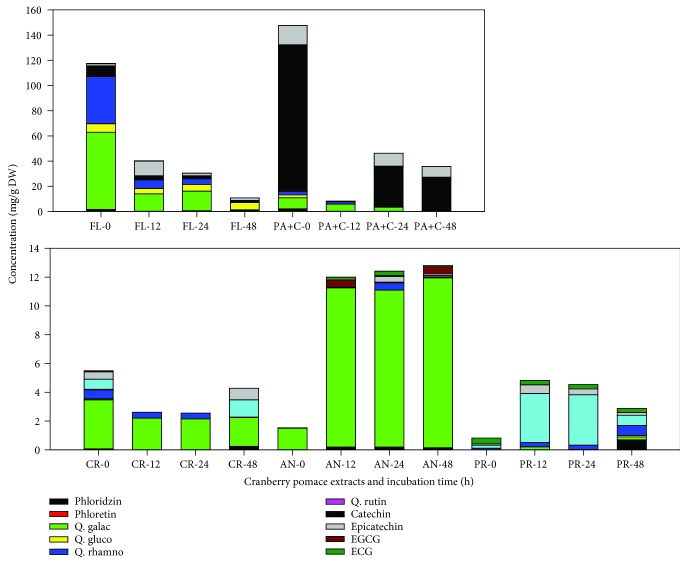
Effects of incubation with *L. rhamnosus* on the degradation of flavonoids present in cranberry pomace fractions. Data presented the mean values of *n* = 3 with respect to medium control. CR, crude; AN: anthocyanin; PR: proanthocyanidin: FL: flavonol; PA+C: phenolic acid+catechin. 0, 12, 24, and 48 are the incubation time (hours).

As *L. rhamnosus* was found to tolerate the presence of all selected CP fractions at concentrations less than 1500 mg/L, our strategy for this study was to stimulate metabolism of different polyphenol-rich CP fractions over a range of time points in order to investigate their biotransformation using *L. rhamnosus*. We used only one bacterial strain because probiotic bacteria and the polyphenol interactions for the production of biotransformed metabolites were the objectives of this study. This approach has been previously followed to study the biotransformation capability of grape seed [20] and wine polyphenols [22]. The incubations resulted in partial to complete depletion of parent compounds with the formation of new products. The results explicitly showed that the catabolic activity of *L. rhamnosus* modified the phenolic profiles of the tested CP extracts (Figures 3 and 4). A drop in concentration was observed in the selected polyphenol-rich media without bacteria, which could be due to their instability (oxidation and epimerization) during incubation in solutions with pH ~5.5.

Major phenolic compounds present in the CP, including flavonols (quercetin galactoside and quercetin rhamnoside), flavan-3-ols (catechins and epicatechins), anthocyanins, and proanthocyanidins, decreased in concentration after incubation with *L. rhamnosus*, while the concentrations of other phenolic acids and aromatic derivatives, including 4-hydroxyphenyl acetic acid, 3-(4-hydroxyphenyl)propionic acid, hydrocinnamic acid, catechol, and pyrogallol, increased. However, the stability of individual compounds also varied between different extracts and also with respect to time of incubation, as reflected from their percentage recovery. Interestingly, succinic acid present in CP fractions was used up by the bacteria in the first 12 h; however, its concentration was found to increase concerning incubation time post 12 h. There were exceptions to this phenomenon with PR fraction exhibiting a sharp increase in succinic acid concentration after 12 h, followed by a continuous decrease. Succinic acid is produced by *Lactobacilli* species when grown in MRS media [23]. Therefore, the data presented in Figure 4 has been corrected with respect to the control.

#### 3.3.1. PR Fraction

The most noticeable increase in metabolite concentration was found in the PR fraction with 18-23-fold increase during 12-48 h incubation time, thereby clearly demonstrating extensive catabolism of their polymeric structure by the *L. rhamnosus*. An increase in catechin (0.78 mg/g), epicatechin (0.3 mg/g), benzoic acid (1.7 mg/g), 4-hydroxyphenylacetic acid (17.5 mg/g), and most importantly, 3-(4-hydroxyphenyl)propionic acid (19.5 mg/g) after 48 h incubation described the bioconversion process. Interestingly, the concentration of protocatechuic acid also increased by 8.5-fold after 48 h of incubation. Most likely, it could have been derived from 3,4-dihydroxyphenylpropionic acid by its decarboxylation and dehydroxylation to 3-hydroxybenzoic and 4-hydroxybenzoic acids catalyzed by the gut microorganisms [24]. Unlike the previous study with *L. plantarum* IFPL935 [20], the concentration of pyrogallol did not change during incubation of PR and PA+C fractions with *L. rhamnosus*. This can be because the CP used in the present study did not contain high amounts of galloylated flavanols as present in grape seed extract used by the previous study. As we know, a majority of the procyanidins in cranberry press cake come with a high degree of polymerization (DP) [25]. Although several of their biological activities have been shown [26], yet they are poorly absorbed relative to their corresponding monomers. Cranberry proanthocyanidins are microbially catabolized to generate low molecular weight derivatives that further undergo phase II metabolism inside the liver before reaching plasma and tissues [27]. Therefore, it is interesting to identify further biologically active components of metabolized proanthocyanidins in order to justify their *in vitro* biological activity.

#### 3.3.2. PA+C Fraction

A decrease in the response was observed for caffeic, ferulic, isoferulic, and benzoic acids. Concurrently with the disappearance of these compounds, the formation of 4-hydroxyphenylacetic acid and catechol, together with a significant increase in the response of hydrocinnamic acid and 3-(4-hydroxyphenyl)propionic acid, was observed. None of these metabolites were found in the incubations of *L. rhamnosus* growth control. Metabolism of ferulic acid is known to result in coumaric acid, which is further decarboxylated to hydroxybenzoic acid, benzoic acid, phenol, and catechol [13, 28]. However, in our case, coumaric acid degraded after 12 h, but its concentration increased again after 24 h and stayed statistically insignificant after 48 h. The formation of hydroxyphenyl propionic acids from C−C double-bond reduction in *m*-coumaric and ferulic acids by other *Lactobacillus* strains including *L. plantarum* has been described previously [29]. Besides, the formation of 3-hydroxyphenylpropionic and benzoic acids from caffeic acid esters also has been established [30]. The formation of catechol from phenolic acids such as protocatechuic acid and caffeic acid can be explained by the decarboxylase action of *Lactobacillus* spp. [29]. Catechin and epicatechin suffered a progressive diminution during incubation and practically 77% and 44% loss of concentration at 48 h, respectively. According to previous studies, the catabolism of epicatechin using human intestinal bacteria, through a series of steps, produces valerolactones [20, 31], which were not analyzed in the present study. However, the appearance of high concentrations of 3-(4-hydroxyphenyl)propionic acid and methyl 3,4,5-trihydroxybenzoate during the incubation of PA+C may suggest the catechin family catabolism through phenylpropionic acids and benzoic acids. In addition, the galloylated monomeric flavan-3-ols (ECG and EGCG), which were low in concentration at the beginning, were also degraded during the incubation.

#### 3.3.3. CR Fraction

To expand the spectrum of phenolic substrates catabolized by *L. rhamnosus*, the present study also investigated its ability through a crude cranberry pomace extract. With respect to the data prior to incubation, the CR fraction showed an increase of total LCMS-based phenolic acids by 1.7-, 2.0-, and 1.8-fold after 12, 24, and 48 h, respectively. Some of the interesting features were the increase in concentrations of hydroxycinnamic acids, including ferulic, caffeic acids, and *p*-coumaric (the later to a lesser extent) after 48 h of incubation. This was in contrast to the previous study by Sánchez-Patán et al. [20] that showed a decrease in concentrations of these compounds, after incubation with *L. plantarum* IFPL935. The concentration of hydroxybenzoic acids, majorly protocatechuic acid, in this case, decreased within the first 24 h to almost half of its concentration, but again increased by more than 10-fold after 48 h. Also, a new compound, 3-(4-hydroxyphenyl)propionic acid, appeared within the first 12 h, together with a significant increase in the responses of 4-hydroxyphenylacetic acid and pyrogallol (present at very low concentration in the beginning), was observed. On the other hand, benzoic acid was found to degrade over time, while succinic acid showed an increase. The formation of hydroxyphenylpropionic acids by *Lactobacillus* species from the C-C double bond reduction of hydroxycinnamic acids has been explained [29]. Owing to anthocyanin metabolism, the concentration of syringic acid went high by 9-fold during the bioconversion.

#### 3.3.4. AN Fraction

The AN fraction was generally less stable and presented large variations in the phenolic acid concentrations from 0 h to 48 h. Hydrolysis of anthocyanin glycosides by enzymes by cleavage of the 3-glycosidic linkage is proposed as the first step within 20 min to 2 h for subsequent bacterial degradation and the formation of a set of new metabolites that have not yet been identified [32, 33]. For that very reason, no aglycones were detected under any of the collection times assayed. The released aglycones formed transitorily could have been degraded into the corresponding phenolic acids emanating from the B ring [32–34]. Concerning the AN fraction, as expected, mainly formation of gallic, syringic, and p-coumaric acids took place. The concentration of syringic acid increased continuously during incubation and went up by almost 10-fold after 48 h. As previously established, syringic acid is the major metabolite of anthocyanins, particularly malvidin glucosides [35]. Incubation of AN fraction with *L. rhamnosus* also yielded pyrogallol, indicating further metabolism such as decarboxylation of the metabolites such as gallic acid. During the first 24 h, p-coumaric acid increased by 23-fold, suggesting the bioconversion of anthocyanins using *L. rhamnosus*. In addition, small amounts of methyl gallate and protocatechuic acid were also produced during incubation. Protocatechuic acid has been identified as the major degradation product of cyanidin-3-*O*-glucoside, after incubation with human feces [34].

Interestingly, the concentration of 4-hydroxyphenylacetic acid spiked in the 12 h incubation by more than 21-fold, which was further reduced to 5.3-fold at the end of 48 h. This reduction could be due to further degradation of 4-hydroxyphenylacetic acid to simpler metabolites such as benzoic acid, which showed a slight increase in its concentration after 48 h. Similar information on the production of phenylacetic acids and hydrated acids has been described earlier by some authors [27, 36–38].

#### 3.3.5. FL Fraction

Most of the quercetin glycosides are not absorbed in the small intestine and deglycosylated under the effect of bacterial *β*-glucosidases and *α*-rhamnosidase in the large intestine. Enzymes of intestinal bacteria not only deglycosylate quercitrin (quercetin-3-*O*-rhamnoside) but also further metabolize it with the break of quercetin heterocycle and formation of phenol acids such as 3,4-dihydroxyphenylacetic, hydroxyphenylacetic, and 3-methoxy-4-hydroxyphenylacetic acids [39]. Similar results were found in this study using *L. rhamnosus* incubation. The concentration of quercetin glycosides went down drastically, with the concurrent increase of metabolite concentrations including 4-hydroxyphenylacetic acid, hydrocinnamic acid, 3-(4-hydroxyphenyl)propionic acid, and protocatechuic acid, along with metabolic pathway end products like benzoic acid. The first 12 h incubation presented a 20-fold increase in protocatechuic concentration, which decreased by 3-fold after 48 h. On the other hand, the concentrations of 4-hydroxyphenylacetic acid and 3-(4-hydroxyphenyl)propionic acid showed a steep increase of 21-35 and 28-33 mg/g DW, respectively. Also, the benzoic acid concentration was found to increase by 1.6-fold, after 48 h incubation. Apart from the quercetin glycosides, the phenolic acid concentration of the FL fraction as well degraded during the bioconversion process.

### 3.4. Qualitative Measurements

**Figure 5:**
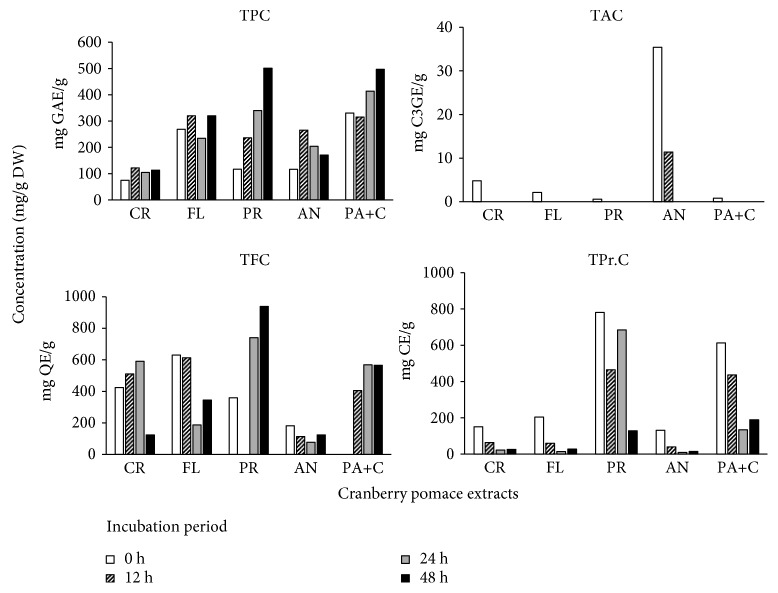
Total phenolic content (TPC), total anthocyanin content (TAC), total proanthocyanidin content (TPr.C), and total flavonoid content (TFC) of cranberry pomace fractions. Data presented the mean values of *n* = 3 with respect to the medium control. CR: crude; AN: anthocyanin; PR: proanthocyanidin: FL: flavonol; PA+C: phenolic acid+catechin.

The data for total phenolic content (TPC), total anthocyanin content (TAC), total proanthocyanidin content (TPr.C), and total flavonoid content (TFC) is represented in Figure 5. The bioconversion process significantly increased the TPC of all bioconverted fractions when compared to the fractions without biotransformation. While there were no similar studies on cranberry at the time of writing, these results were in line with a report that showed that fermentation of *C. lanceolata* increased the TPC in comparison to extracts without any fermentation [40]. The surge in the TPC for both PA+C and PR fractions was remarkable at 48 h as compared to 0 h. Fractions AN, PR, FL, and PA+C increased about 2-4 times in their TPC than the CR extract. A similar trend was observed for TFC, except for the AN fraction. On the contrary, the TAC and TPr.C degraded significantly during the bioconversion. Again, this is possibly due to the catabolism of these fractions into their respective metabolites. Interestingly, after initial degradation at 12 h, the TPr.C was noticed to increase in PR fraction at 24 h. This could be because of initial catabolism of proanthocyanidin polymers into monomeric flavan-3-ols that were read as catechin equivalents.

### 3.5. Biotransformed CP Polyphenol Fractions Cytotoxic to Cancer Cells at Lower Concentrations than Their Parent Fractions

#### 3.5.1. Screening of CP Fractions and Their Metabolites on the Viability of HepG2 Cells

As a cell model of anticancer, we have used a widely investigated HCC HepG2 cells. This cell line can provide us an insight into the chemotherapeutic potential of the bioconverted metabolites for controlling malignant hepatocyte growth of liver cancer. The liver is an important organ which is the pivotal site for toxicity of xenobiotics, drugs, and oxidative stress [41]. Also, the liver is important for metabolic activation or inactivation of potentially antioxidative or other biologically active substances. We first sought to standardize the optimum concentration of CP fractions to inhibit the proliferation of the HCC cell line. The HepG2 cells were administered with increasing concentrations (1, 10, 50, 100, 250, and 500 *μ*g/mL) of sorafenib or CP fractions before and after 24 h of bioconversion using *L. rhamnosus*, and cell viability was evaluated at 24 and 48 h after treatment. The obtained data resulted in a time- and concentration-dependent decrease in cell viability (Figure S1). In general, the CP metabolite extracts after 24 h bioconversion were more effective in inhibiting HepG2 cell proliferation than their parent counterparts (0 h), except for anthocyanin-rich fraction. Among all the treatments given, PR 24 h showed the most potent antiproliferation activity, by displaying no viability at 100 *μ*g/mL and displaying an IC_50_ value of 48 and 20 *μ*g/mL (Table 3). This was followed by PA 24 h exhibiting 37% cell viability at 100 *μ*g/mL which was not significantly different from the 28% viability demonstrated by the drug sorafenib. Therefore, it is evident that the *L. rhamnosus* bioconversion of cranberry pomace proanthocyanidins (PR 24 h) produces metabolites which are biologically more active than their intact forms. This high biological activity could be associated with their low molecular weight phenolic acid structures and monomeric flavan-3-ols, which can penetrate through the cell wall efficiently [8]. This is in agreement with a previous study that showed water-soluble phenolic extracts of cranberry and its products (mainly low molecular weight phenolic acids and their derivatives) effectively inhibited the proliferation of HT-29 and LS-513 colon cancer cell lines [42].

The other CP fractions including CR, AN, and FL showed significantly lower antiproliferation activity than the above-discussed PR and PA extracts. Therefore, these three fractions were excluded from a further study. The low biological activity of anthocyanin and flavonol-rich fractions can be attributed to their glycosylation, which could render the molecules more water-soluble but less reactive towards free radicals and metals, diminishing their antioxidant activity [5]. Additional experimentation was carried out using proanthocyanidins fraction before and after 24 h bioconversion and compared to two of its most prominent metabolites formed after 24 h, i.e., 3-(4-hydroxyphenyl)propionic acid and 4-hydroxyphenylacetic acid, along with sorafenib. The results obtained for the MTS assay revealed that at 100 *μ*g/mL, the PR 24 h showed the highest antiproliferative activity compared to all the test compounds. The PR 24 h showed significantly higher (*p* < 0.05) activity than the prescribed drug sorafenib at the tested concentration. The two metabolites tested (3-(4-hydroxyphenyl)propionic acid and 4-hydroxyphenylacetic acid) did not show any significant activity at the concentrations between 10 and 100 *μ*g/mL. Therefore, it can be speculated that the observed antiproliferative activity of PR 24 h could be due to the combined effects of several bioactive metabolites present in the fractions and this synergistic action can be better than any single compound. Further, a concentration of 50 and 100 *μ*g/mL was selected for PR fractions for further studies.

#### 3.5.2. PR 24 h Led to Metabolic Depletion of ATP in HepG2 Cells

Previous studies have shown that mitochondria play an essential role in the regulation of apoptosis in cells. After initial screening of CP fractions against HepG2 cell proliferation, we selected PR 24 h as the most potent candidate for further analyses based on its IC_50_ values. Two of its most abundant metabolites, i.e., 3-(4-hydroxyphenyl)propionic acid and 4-hydroxyphenylacetic acid and sorafenib, were compared for their total ATP-depleting activity. As shown in Table 4 and Figure S2, the cellular ATP activity decreased with the increasing concentration of PR 24 h and sorafenib and both displayed comparable effects at 100 *μ*g/mL (*p* ≤ 0.05) *in vitro*. However, at the lower concentrations of 10-20 *μ*g/mL, the ATP activity displayed by sorafenib was half than that exhibited by PR 24 h. Surprisingly, the data suggested that 3-(4-hydroxyphenyl)propionic acid and 4-hydroxyphenylacetic acid stimulated cellular proliferation at the lower concentrations with data points close to 100% cell viability. Given the cellular ATP depletion by PR 24 h, it could be speculated that this metabolic extract could activate Apaf-1 function and mitochondria-controlled apoptosis, as suggested by Ferrari et al. [43].

#### 3.5.3. Induction of Caspase-3 by PR 24 h

Activation of caspase-3 is a key step in multiple apoptotic cell death pathways. Caspases contribute to apoptosis by cleavage of various cellular substrates and modulate both cellular integrity and cell cycle through chromatin condensation, loss of cell adhesion, cell shrinkage, membrane blebbing, DNA fragmentation, and formation of apoptotic bodies that are engulfed by phagocytes. Therefore, we examined the effect of PR 0 h, PR 24 h, and the two selected metabolites for stimulating caspase-3 activation. Enzymatic activity of caspase-3 changed after 24 h of incubation with the anticancer candidates using all treatments, except the control (Figure S3). The assay also revealed that with compounds' treatment the proportion of caspase enzyme-activated cells increased time-dependently, indicating the activation of these enzymes in the cells. In line with a previous study, sorafenib triggered the expression of caspase-3 enzyme among all the assayed compounds [44], which was significantly higher than all the assayed extracts/compounds. The CP fraction PR 24 h displayed the highest caspase-3 release at both 50 *μ*g/mL and 100 *μ*g/mL in comparison to PR 0 h and the two selected pure compounds. The activity of the caspase was threefold higher at 50 *μ*g/mL but decreased to less than two times at a concentration of 100 *μ*g/mL. Nevertheless, it can be suggested that cell death was not much influenced by the activation of effected caspases 3 and 7. However, further studies are required to identify other target pathways responsible for the cytotoxic activity of the CP fraction PR 24 h. Moreover, it is also interesting to investigate the ability of probiotic metabolites of cranberry proanthocyanins in the prevention of carcinogen-induced HCC in response to recently reported reduction of chemical-induced DNA damage by some common microbial metabolites of proanthocyanidins [45].

## 4. Conclusion

Despite the fast-growing research on fruit polyphenols and their potential health benefits, poor bioavailability of polymeric proanthocyanidins endorses the development of biotransformation approaches. While there have been reports on the improvement in biological activities of polyphenols upon their probiotic biotransformation, to our knowledge, this is the first study that investigated the bioconversion of cranberry proanthocyanidins and the effect of their metabolites on HCC cells *in vitro*. Biotransformation of proanthocyanidins into low molecular weight phenolic metabolites displayed cytotoxic activity against HepG2 cells at physiologically relevant concentrations. Further analyses revealed the involvement of mitochondrial ATP depletion and partial caspase 3/7 activation that attributed to their potential anticancer activity.

## Figures and Tables

**Table 1:** Changes in pH of different cranberry pomace (CP) extracts during bioconversion with *L. rhamnosus*.

Time	Control	Type of extract				
		CR	FL+DHC	PA+C	AN	PR
0 h	5.84	5.84	5.86	5.84	5.85	5.85
24 h	3.81	3.85	3.83	3.63	3.78	3.59
48 h	3.32	3.40	3.33	3.22	3.41	3.40

CR: crude; FL+DHC: flavonol+dihydrochalcone; PA+C: phenolic acid+catechin; AN: anthocyanin; PR: proanthocyanidin. Control contains *L. rhamnosus* and media with no CP extracts added.

**Table 2:** The percent growth of *L. rhamnosus* in the presence of various cranberry fractions with a comparison to the control.

CP extract	Concentration (mg/L)			
	250	500	1000	1500
CR	122.9 ± 2.0	110 ± 4.4	94.2 ± 5.2	77.1 ± 4.0
AN	106.6 ± 1.0	92.9 ± 1.4	74.3 ± 3.9	62.5 ± 5.7
PR	120.0 ± 2.5	107.5 ± 2.5	95.0 ± 4.5	86.3 ± 4.5
FL+DHC	125.2 ± 0.3	103.8 ± 3.8	91.3 ± 3.8	76.7 ± 3.8
PA+C	115.0 ± 7.5	100.8 ± 6.4	90.0 ± 2.5	68.8 ± 3.7

The growth of *L. rhamnosus* was measured by optical density at 625 nm. The control does not contain any fractions but the growth medium and *L. rhamnosus*. CR: crude; FL+DHC: flavonol+dihydrochalcone; PA+C: phenolic acid+catechin; AN: anthocyanin; PR: proanthocyanin.

**Table 3:** The IC_50_ (mg/L) for cytotoxic effect in HepG2 cells of various cranberry fractions before and after bioconversion.

Assay	Source	Bioconversion	24 h	48 h
MTS	CR	Before	>500	>500
		After	340.9	319.6
	AN	Before	130.1	121.3
		After	241.7	266.7
	FL+DHC	Before	206.8	223.0
		After	115.8	102.6
	PA+C	Before	58.9	47.8
		After	41.2	36.4
	PR	Before	164.9	107.2
		After	47.8	20.1
	4-Hydroxyphenylacetic acid		183.5	136.9
	3-(4-Hydroxyphenyl)propionic acid		219.0	153.6

The cell viability was measured using the MTS assay after incubation of HepG2 cells with test extracts for either 24 or 48 h. CR: crude; FL+DHC: flavonol+dihydrochalcone; PA+C: phenolic acid+catechin; AN: anthocyanin; PR: proanthocyanin. Control contains *L. rhamnosus* and growth medium with no CP extracts added.

**Table 4:** Total ATP content of HepG2 cells after 24 h when treated with cranberry extracts before and after bioconversion using *L. rhamnosus*.

Treatment	Bioconversion (24 h)	Total ATP content (mg/L)
PR	Before	105.6
PR	After	3.7
4-Hydroxyphenylacetic acid		431.3
3-(4-Hydroxyphenyl)propionic acid		>500
Sorafenib		0.01

PR: proanthocyanin.

## Data Availability

All the data are included in the manuscript and under supplementary data.

## Supplementary Materials

Supplementary Materials Figure S1: percentage viability of HEPG2 cells as affected by the different concentrations of cranberry pomace polyphenols before and after bioconversion (24 h). Cell viability was analyzed by the MTS assay. Data (*mean* ± *SE*, *n* = 5) are expressed as percentages of the MTS level detected in untreated control cells. Figure S2: ATP activity of HEPG2 cells as affected by the different concentrations of cranberry pomace polyphenols, pure compounds, and a drug after 24 h. Figure S3: caspase-3/7 activity of HEPG2 cells as affected by the different concentrations of cranberry pomace polyphenols, pure compounds, and a drug after 24 h. 4-HPAA: 4-hydroxyphenylacetic acid; 3-4HPPA: 3-4-hydroxyphenylpropionic acid.
