# Supplementary material for: Biotransformation of Cranberry Proanthocyanidins to Probiotic Metabolites by Lactobacillus rhamnosus Enhances Their Anticancer Activity in HepG2 Cells In Vitro
Source: Oxid Med Cell Longev. 2019 Jun 17;2019:4750795. doi: 10.1155/2019/4750795 (PMC6604286; doi:10.1155/2019/4750795)
Supplement: Supplementary Materials — Figure S1: percentage viability of HEPG2 cells as affected by the different concentrations of cranberry pomace polyphenols before and after bioconversion (24 h). Cell viability was analyzed by the MTS assay. Data (mean ± SE, n = 5) are expressed as percentages of the MTS level detected in untreated control cells. Figure S2: ATP activity of HEPG2 cells as affected by the different concentrations of cranberry pomace polyphenols, pure compounds, and a drug after 24 h. Figure S3: caspase-3/7 activity of HEPG2 cells as affected by the different concentrations of cranberry pomace polyphenols, pure compounds, and a drug after 24 h. 4-HPAA: 4-hydroxyphenylacetic acid; 3-4HPPA: 3-4-hydroxyphenylpropionic acid. [file 4750795.f1.pdf]

Supplementary Data

Figure S1: Percentage viability of HEPG2 cells as affected by the different concentration of cranberry pomace polyphenols before and after bio-conversion (24 h).

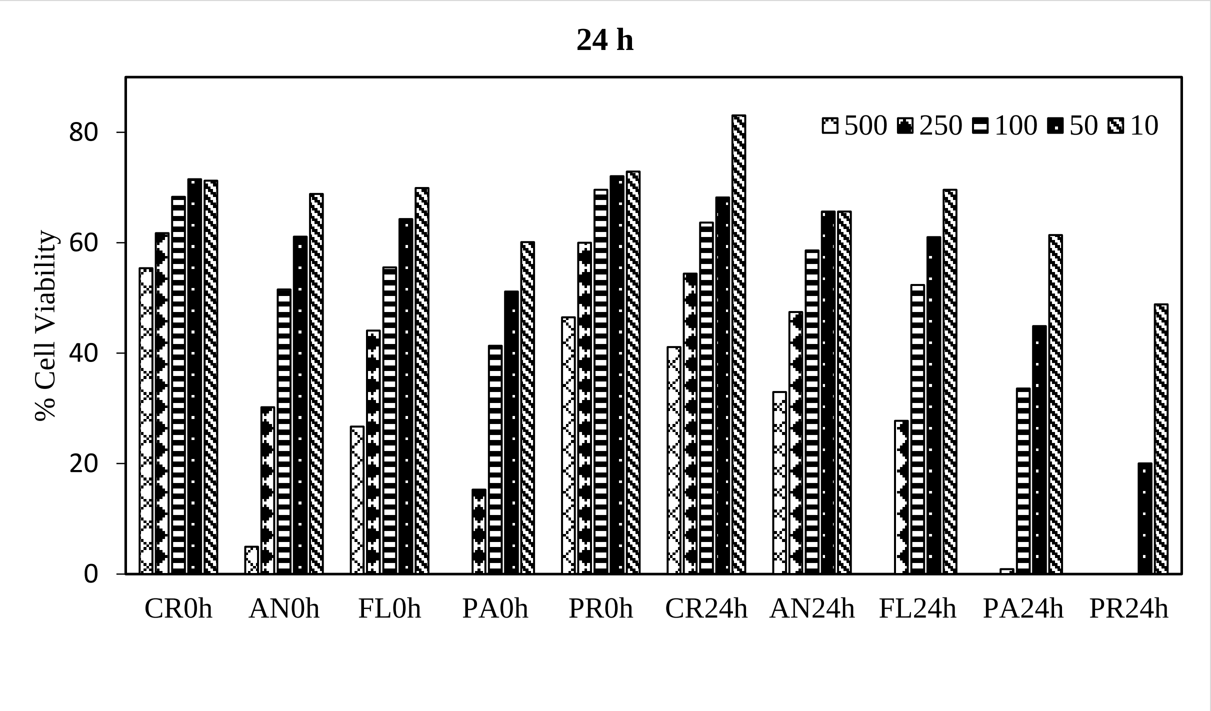

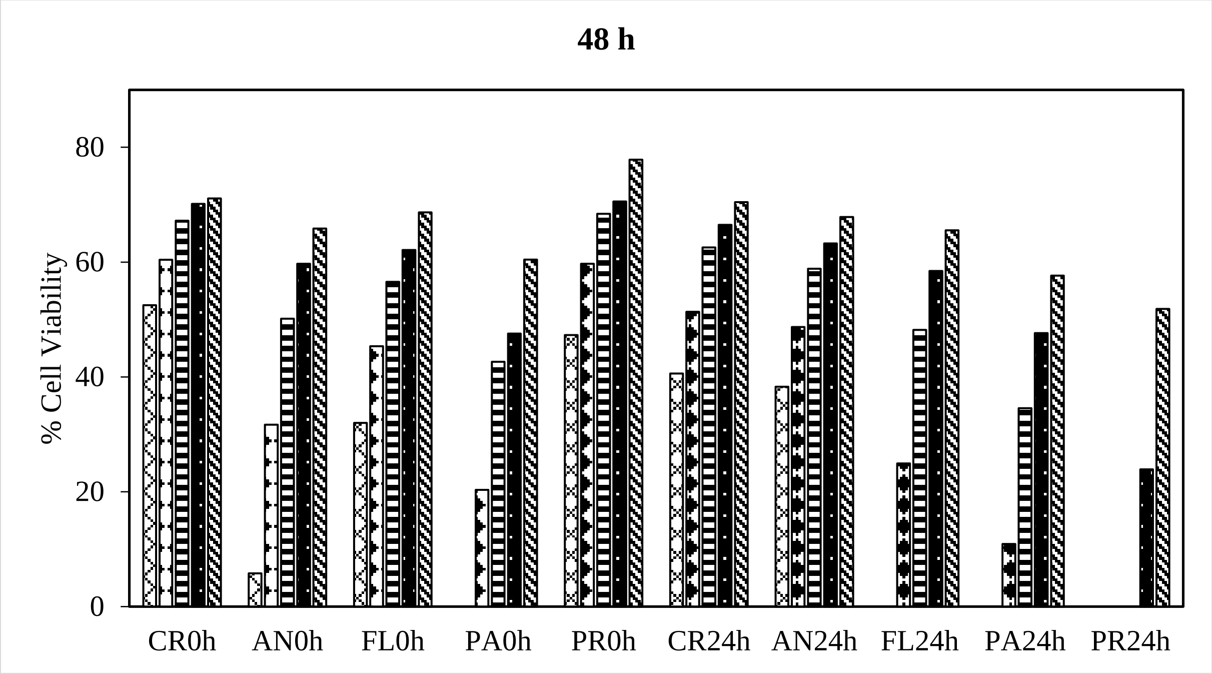

Cell viability was analyzed by the MTS assay. Data (mean ± SE, *n* = 5) are expressed as percentages of the MTS level detected in untreated control cells.

Figure S2: ATP activity of HEPG2 cells as affected by the different concentration of cranberry pomace polyphenols, pure compounds, and drug after 24 h.

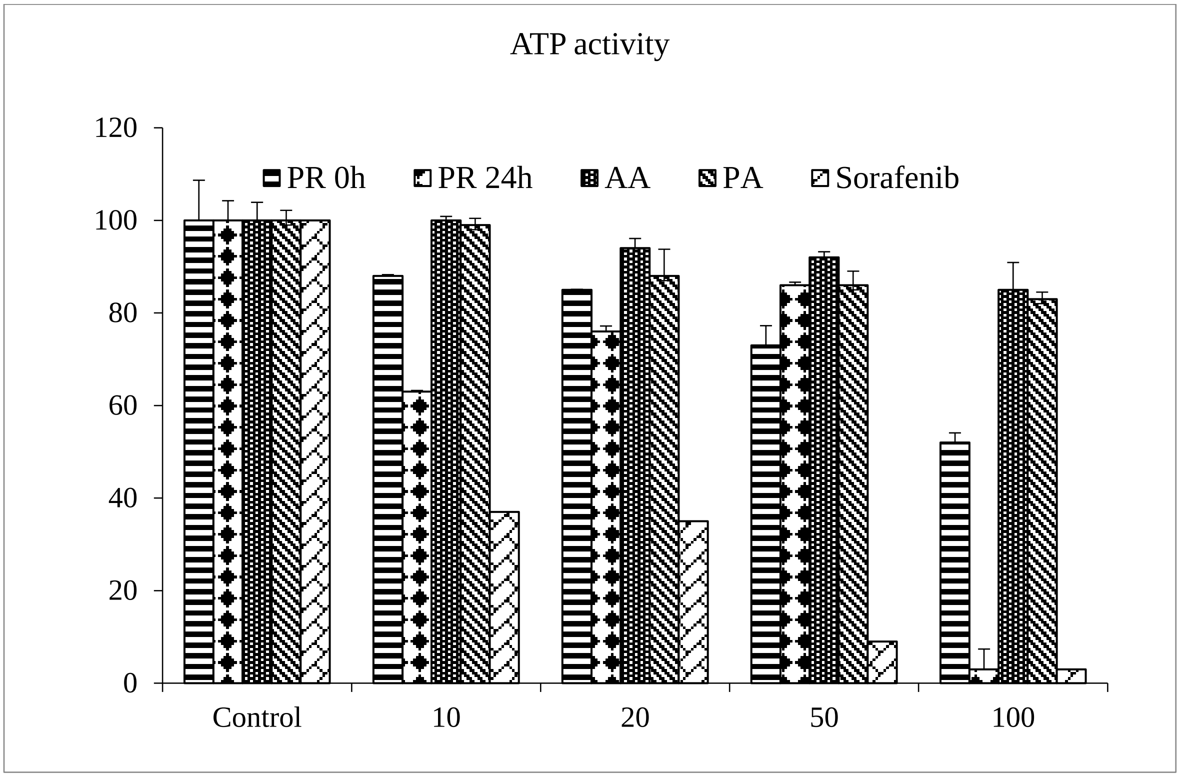

Figure S3: Caspase-3/7 activity of HEPG2 cells as affected by the different concentration of cranberry pomace polyphenols, pure compounds, and drug after 24 h.

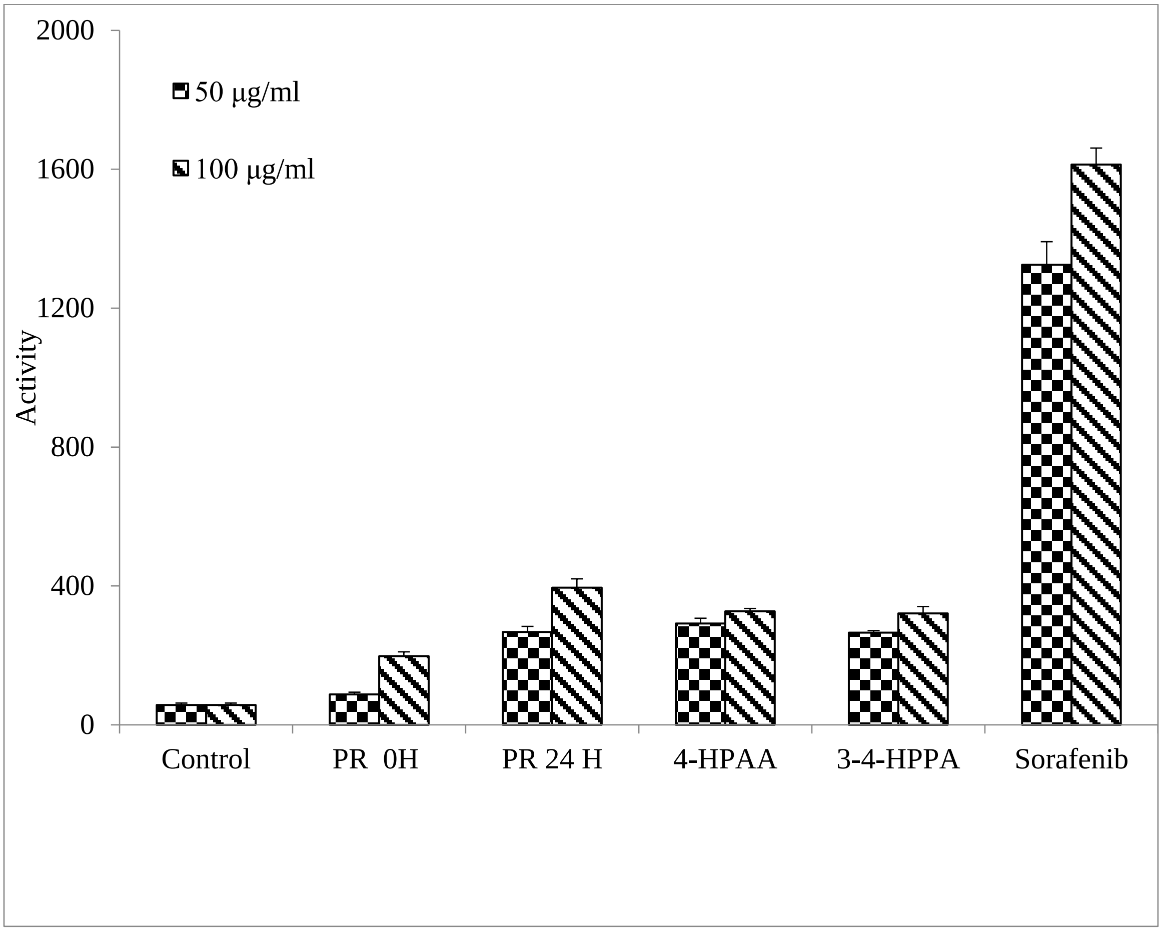

4-HPAA: 4-hydroxyphenylacetic acid; 3-4HPPA: 3-4-hydroxyphenylpropionic acid
